# Impact of prophylactic antibiotic duration on surgical site infection rate in neonatal surgery: a multicenter retrospective observational study

**DOI:** 10.1038/s41372-025-02400-3

**Published:** 2025-08-27

**Authors:** Tomohiro Sunouchi, Jun Fujishiro, Koji Oba, Tetsuya Ishimaru, Chikara Ogimi, Hiroshi Kawashima, Akira Nishi, Kenichi Maruyama, Kiyoshi Tanaka, Hajime Takayasu, Yujiro Tanaka, Kazuko Obana, Ryu Yoneda, Akio Ishiguro, Yutaka Matsuyama

**Affiliations:** 1 Department of Pediatric Surgery, The University of Tokyo Hospital, Tokyo, Japan; 2 Department of Biostatistics, School of Public Health, Graduate School of Medicine, The University of Tokyo, Tokyo, Japan; 3 Division of Surgery, Department of Surgical Specialties, National Center for Child Health and Development, Tokyo, Japan; 4 Division of Infectious Diseases, Department of Medical Subspecialties, National Center for Child Health and Development, Tokyo, Japan; 5 Department of Surgery, Saitama Children’s Medical Center, Saitama, Japan; 6 Department of Surgery, Gunma Children’s Medical Center, Gunma, Japan; 7 Department of Neonatology, Gunma Children’s Medical Center, Gunma, Japan; 8 Division of Pediatric Surgery, Research and Development Center for New Medical Frontiers, Kitasato University School of Medicine, Kanagawa, Japan; 9 Department of General-Pediatric Hepato Biliary Pancreatic Surgery, Kitasato University School of Medicine, Kanagawa, Japan; 10 Department of Pediatric Surgery, Saitama Medical University Hospital, Saitama, Japan; 11 Department of Pediatric Surgery, Japanese Red Cross Medical Center, Tokyo, Japan; 12 Department of Infection Control and Prevention, The University of Tokyo Hospital, Tokyo, Japan; 13 Perinatal Maternal and Child Medical Center, The University of Tokyo Hospital, Tokyo, Japan

**Keywords:** Risk factors, Infection

## Abstract

**Objective:**

To evaluate the relationship between the incidence of surgical site infections (SSIs) and the duration of perioperative antibiotic prophylaxis (PAP) in neonatal surgery, and to identify risk factors for SSIs in neonates.

**Methods:**

Eligible patients were neonates who underwent surgical procedures—primarily in the respiratory and gastrointestinal fields—between January 2014 and December 2023 at seven institutions. All data were retrospectively retrieved from electronic patient records. We estimated the risk difference using a modified least-squares regression model.

**Results:**

Of the 983 patients included, 91 (9%) developed SSIs. A total of 735 patients (75%) received PAP for >24 h. There was no significant difference in risk when PAP duration was <24 h compared with ≥24 h. Independent risk factors for SSIs were an operative time exceeding 120 min, past surgical history, and open surgery.

**Conclusion:**

In neonatal surgery, a short duration (<24 h) of PAP may not increase the risk of SSI.

Perioperative antibiotic prophylaxis (PAP) is essential for the prevention of surgical site infections (SSIs). In adult surgery, sufficient evidence-based guidelines recommend short-term administration of PAP, i.e., discontinuing PAP within 24 h [1, 2]. In pediatric surgery, however, there was historically a lack of evidence, and guidelines for adults were often extrapolated or PAP was administered empirically. As a result, PAP tended to be prolonged. Evidence for pediatric patients has recently accumulated [3–6], and short-term administration is now also recommended and gradually becoming more widespread [7].

The situation in neonatal surgery is different. Some studies [8–10] suggest that short-term administration of PAP might be safe and effective for neonates as well as older children, and one of the few guidelines [11] recommends short-term administration of PAP for neonates. However, the latest studies reveal that PAP for neonates is still being administered for a long duration [12, 13].

Prolonged antibiotic administration is well known to be associated with many adverse effects, such as nephrotoxicity, *Clostridium difficile* infection [14], and multidrug-resistant organisms [15]. In recent years, antibiotic use in early childhood has also been implicated in the development of several diseases, such as asthma and inflammatory bowel disease, through alterations in the gut flora [16, 17]. This makes it an even more important issue for neonates, who have a long life ahead of them.

Unfortunately, this trend of long-term administration of PAP for neonates has been noted for 30 years [18], and the situation has not changed [12, 13]. The main reason for this is thought to be the weak level of evidence regarding PAP in neonates. There are no randomized controlled trials and only a few observational studies on PAP in this population. Conducting randomized controlled trials in neonatal surgery is challenging because of ethical concerns, the wide variety of comorbidities, and the small number of cases. Additionally, neonates often receive therapeutic antibiotics for hospital-acquired infections during the perioperative period because they are highly vulnerable to infection due to their immature immune systems [11]. These factors make it difficult to evaluate the efficacy of PAP in preventing SSIs in neonatal surgery.

The aims of this study were to evaluate the relationship between the incidence of SSIs and the duration of PAP in neonatal surgery and to identify risk factors for SSIs in neonates. To achieve these goals, we conducted a multi-institutional retrospective observational study involving several high-volume pediatric centers in Japan and collected detailed data on antibiotic usage from medical records.

## Methods

### Study design

This multi-institutional retrospective observational study was conducted at seven institutions: The University of Tokyo Hospital, National Center for Child Health and Development, Saitama Children’s Medical Center, Gunma Children’s Medical Center, Saitama Medical University Hospital, Kitasato University Hospital, and Japanese Red Cross Medical Center.

### Patients

Eligible patients were children who underwent surgical procedures—mainly in the respiratory and gastrointestinal fields—during the neonatal period (within 28 days of birth) between 1 January 2014 and 31 December 2023 at the seven participating institutions. Patients were excluded if surgery was performed for peritonitis (due to intestinal perforation, necrotizing enterocolitis, or other causes), gastroschisis, omphalocele, biliary atresia, or myelomeningocele because these conditions inherently require prolonged perioperative antibiotic administration [19]. Patients who developed or were suspected of having a hospital-acquired infection other than SSI—such as urinary tract infection, pneumonia, or catheter-related bloodstream infection—after surgery and received therapeutic antibiotics were also excluded. Additionally, SSI was defined as occurring within 30 days postoperatively, and cases that could not be followed for the full 30 days were excluded from the analysis.

### Data

All data were retrieved from electronic patient records. The variables assessed included sex, birth weight, gestational age at birth, birth place (in-hospital/out-of-hospital), mode of delivery, Apgar score (1 min/5 min), presence of underlying diseases such as chromosomal abnormalities or malformation syndromes, surgical diagnosis, surgical procedure, age and weight at surgery, surgical duration, American Society of Anesthesiologists (ASA) classification, wound class, past surgical history, SSI (occurrence, site, and day of occurrence since surgery), preoperative colonization of methicillin-resistant *Staphylococcus aureus*, and PAP (type and duration).

The duration of PAP was defined as the period from the start of the operation to the last administration of PAP. For patients with SSI, the duration of PAP was counted up to the day before the onset of SSI, even if antibiotics were continued for treatment of the infection.

The definitions of SSI and wound class followed the Centers for Disease Control and Prevention (CDC) manual [20]. In this classification, all small bowel operations—with or without opening the intestinal lumen (e.g., Ladd procedure and surgery for adhesive small bowel obstruction)—were classified as clean-contaminated. Operations with a dirty/infected wound class were excluded from the study because of the need for therapeutic antibiotic administration.

### Statistical analysis

For descriptive statistics, continuous variables are presented as medians and interquartile ranges, while categorical variables are presented as frequencies and percentages.

To evaluate the association between the incidence of SSIs and the duration of PAP, we estimated the risk difference using a modified least-squares regression model and calculated 95% confidence intervals and *P* values [21]. The following variables—identified from previous studies and clinical judgment as potential risk factors—were included in the multivariable analysis: duration of PAP (<24 h, including no PAP, vs. ≥24 h), premature birth (<37 weeks vs. ≥37 weeks), Apgar score at 5 min (<4 vs. ≥4), age at operation (0 day vs. 1–2 days vs. ≥3 days), weight at surgery (<1500 g vs. ≥1500 g), operative time (<120 min vs. ≥120 min), ASA classification (<3 vs. ≥3), surgical approach (surface vs. open vs. thoracoscopy/laparoscopy), wound class (clean vs. clean-contaminated), and past surgical history (yes vs. no). Cases with missing variables were excluded from the multivariable analysis.

In addition, to adjust for two further confounders—institutions and surgical procedures—inverse probability weighting (IPW) using propensity scores was applied to prevent overfitting in the multivariable model, given the limited number of SSIs relative to the number of explanatory variables. The propensity scores were estimated using a logistic regression model that included key covariates from the primary analysis, along with institutions and surgical procedures. Surgical approach and wound class were excluded from the propensity score model because of their strong correlation with surgical procedures; this avoided multicollinearity and improved model stability. To assess the balance of confounders after weighting, a standardized mean difference plot was visually inspected, with a standardized mean difference of <0.2 considered indicative of good balance.

Statistical analyses were performed using JMP^®^ Pro version 17.2.0 and SAS version 9.4 (SAS Institute Inc., Cary, NC, USA).

## Results

### Cohort description

**Fig. 1:**
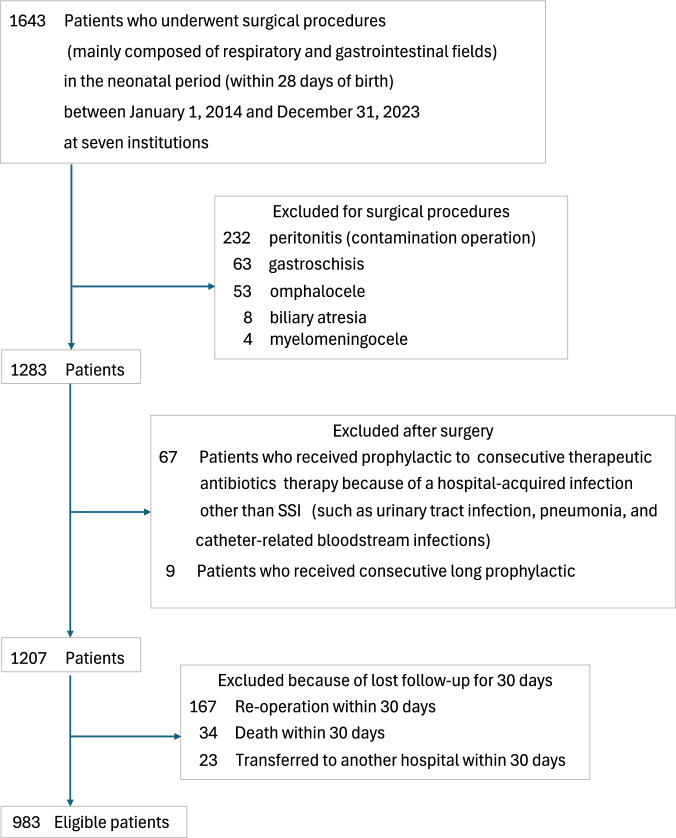
Assembly of study cohort. SSI surgical site infection.

In total, 1643 neonates underwent surgical procedures during the study period at the 7 institutions. Of these, 660 patients were excluded based on the exclusion criteria, resulting in a final study population of 983 patients (Fig. 1). Among the excluded patients, 34 deaths occurred within 30 days, but none were attributed to SSI.

### Incidence of SSIs and duration of PAP

The background characteristics of all 983 patients, as well as those in the No-SSI and SSI subgroups, are shown in Table 1. A total of 91 patients (9%) developed SSIs, with 79 cases (87%) classified as superficial SSIs. The median day of SSI occurrence was 10 days [6–20 days] postoperatively (Supplement 1).

**Table 1:** Demographics and perioperative variables compared with respect to SSI.

	Total (*n* = 983)	No-SSI (*n* = 892)	SSI (*n* = 91)
Hospital, *n* (%)			
A	236 (24%)	205 (23%)	31 (34%)
B	200 (20%)	178 (20%)	13 (14%)
C	191 (19%)	178 (20%)	22 (24%)
D	121 (12%)	114 (13%)	7 (8%)
E	82 (8%)	77 (9%)	5 (5%)
F	77 (8%)	75 (8%)	2 (2%)
G	76 (8%)	65 (7%)	11 (12%)
Sex, *n* (%)			
Male	556 (57%)	509 (57%)	47 (52%)
Female	427 (43%)	383 (43%)	44 (48%)
Gestational age at birth (weeks, days), median [IQR]	38w1d [36w5d-39w1d]	38w1d [36w5d-39w1d]	38w0d [36w5d-39w1d]
Premature birth (<37 weeks), *n* (%)	273 (28%)	244 (28%)	29 (32%)
Birth weight, g, median [IQR]	2690 [2276–3058]	2690 [2272–3054]	2686 [2288–3174]
Very low birth weight (<1500 g), *n* (%)	45 (5%)	42 (5%)	3 (3%)
Birth place, *n* (%)			
in-hospital	577 (59%)	516 (58%)	61 (67%)
out-of-hospital	401 (41%)	371 (42%)	30 (33%)
missing	5	5	0
Mode of delivery, *n* (%)			
Vaginal	520 (54%)	475 (55%)	45 (51%)
Cesarean section	438 (46%)	394 (45%)	44 (49%)
missing	25	23	2
Apgar score at 1 min, *n* (%)			
<4	115 (13%)	102 (12%)	13 (16%)
4–6	167 (18%)	153 (18%)	14 (17%)
>6	634 (69%)	578 (69%)	56 (67%)
missing	67	59	8
Apgar score at 5 min, *n* (%)			
<4	59 (6%)	51 (6%)	8 (10%)
4–6	129 (14%)	117 (14%)	12 (14%)
>6	722 (79%)	659 (80%)	63 (76%)
missing	73	65	8
Underlying diseases, *n* (%)			
13 trisomy	4 (0.4%)	4 (0.4%)	0 (0%)
18 trisomy	23 (2%)	22 (2%)	1 (1%)
21 trisomy	67 (7%)	61 (7%)	6 (7%)
Malformation syndrome	57 (6%)	50 (6%)	7 (8%)
Operative approach, *n* (%)			
Open	759 (77%)	677 (76%)	82 (90%)
Laparoscopy/Thoracoscopy	94 (10%)	87 (10%)	7 (8%)
Body surface	130 (13%)	128 (14%)	2 (2%)
Day of life at operation	2 [1–8]	2 [1–8]	3 [1–12]
0 day	86 (9%)	77 (9%)	9 (10%)
1–2 day	438 (45%)	402 (45%)	36 (40%)
>2 day	459 (47%)	413 (46%)	46 (51%)
Weight at surgery, g, median [IQR]	2668 [2250–3000]	2670 [2250–3000]	2620 [2248–3000]
<1500	50 (5%)	47 (5%)	3 (3%)
Surgical duration, min, median [IQR]	103 [62–150]	99 [61–147]	129 [76–186]
≧120, <180	256 (26%)	226 (25%)	30 (33%)
≧180	139 (14%)	116 (13%)	23 (25%)
ASA classification, *n* (%)			
ASA 1	61 (6%)	56 (6%)	5 (5%)
ASA 2	412 (42%)	376 (42%)	36 (40%)
ASA 3	414 (42%)	377 (42%)	37 (41%)
ASA 4	93 (9%)	80 (9%)	13 (14%)
ASA 5	3 (0.3%)	3 (0.3%)	0 (0%)
Wound class, *n* (%)			
Clean	185 (19%)	168 (19%)	17 (19%)
Clean-contaminated	798 (81%)	724 (81%)	74 (81%)
Past surgical history	64 (7%)	50 (6%)	14 (15%)
Preoperative antibiotic administration	322 (33%)	294 (33%)	28 (31%)
Preoperative MRSA carriers	9 (1%)	8 (1%)	1 (1%)

*SSI* Surgical Site Infection, *IQR* interquartile range, *ASA* American Society of Anesthesiologists, *MRSA* methicillin-resistant Staphylococcus aureus.

Details of PAP administration are presented in Table 2 and Supplement 2. A total of 950 patients (97%) received PAP, with a median duration of 3 days [2–4 days]. Of these, 735 patients (75%) received PAP for more than 24 h. First- and second-generation cephalosporins were used in most cases, at rates of 35% and 41%, respectively.

**Table 2:** Duration and type of PAP compared with respect to SSI.

	Total (*n* = 983)	No-SSI (*n* = 892)	SSI (*n* = 91)
Perioperative antibiotic administration			
Antibiotics given at operation, *n* (%)	950 (97%)	861 (97%)	89 (98%)
Duration of antibiotics, days, median [IQR]	3 [2–4]	3 [2–4]	3 [2–4]
<24 h, *n* (%)	248 (25%)	231 (26%)	17 (19%)
≧24 h, *n* (%)	735 (75%)	661 (74%)	74 (81%)
Type of perioperative antibiotics, *n* (%)			
Cefmetazole	403 (41%)	368 (41%)	35 (38%)
Cefazolin	346 (35%)	307 (34%)	39 (43%)
Ampicillin	67 (7%)	61 (7%)	6 (7%)
Ampicillin+Amikacin	47 (5%)	45 (5%)	2 (2%)
Cefotaxime	32 (3%)	29 (3%)	3 (3%)
Ampicillin/Sulbactam	21 (2%)	19 (2%)	2 (2%)
no-antibiotics	33 (3%)	31 (3%)	2 (3%)

*PAP* Perioperative Antibiotic Prophylaxis, *SSI* Surgical Site Infection, *IQR* interquartile range.

**Fig. 2:**
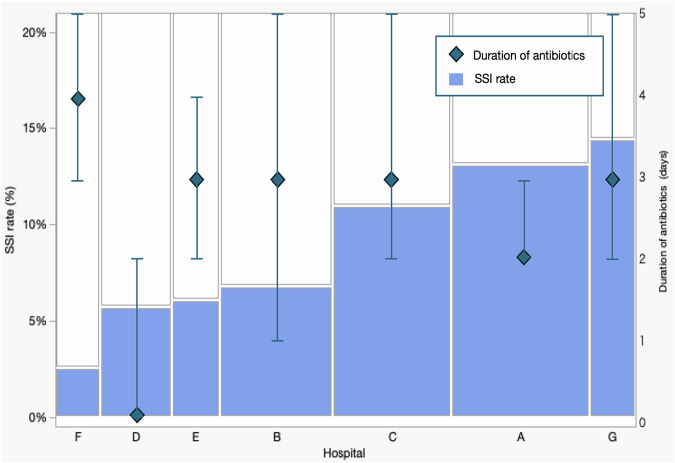
Variation in SSI rate and duration of perioperative antibiotic prophylaxis across seven hospitals. The duration of perioperative antibiotic prophylaxis and the SSI rate at the seven participating hospitals are shown. Diamonds indicate the median duration of antibiotics, with vertical bars showing interquartile ranges. The width of each box represents the number of cases at that institution. SSI surgical site infection.

Supplement 3 and Supplement 4 show trends over time in PAP duration and SSI rate, respectively. Although there was a gradual trend toward shorter PAP duration, no clear trend was observed in the SSI rate. Figure 2 illustrates the relationship between PAP duration and SSI rate across institutions. There was no clear association between the median duration of PAP and the SSI rate among the institutions.

### Multivariable regression analysis: relationship between incidence of SSIs and duration of PAP

Table 3 shows the results of the multivariable analysis using a modified least-squares regression model to estimate risk differences for factors associated with the incidence of SSIs. Seventy-five patients with missing data on risk factors (including eight patients with SSI) were excluded from the analysis.

**Table 3:** Multivariable regression model for the incidence of SSIs.

		Risk difference	95% CI	*P*
Duration of antibiotics	<24 h	−2.7%	(−6.6%–1.3%)	0.187
	≧24 h	Reference		
Sex	Male	−1.5%	(−5.4%–2.4%)	0.444
	Female	Reference		
Premature birth	<37 weeks	0.7%	(−4.4%–5.9%)	0.780
	≥37 weeks	Reference		
Apgar score at 5 min	<4	3.1%	(−6.3%–12.4%)	0.517
	≧5	Reference		
Operative approach	Open	5.9%	(2.0%–9.8%)	0.003
	Laparoscopy/Thoracoscopy	2.4%	(−4.3%–9.2%)	0.480
	Body surface	Reference		
Day of life at operation	0 day	1.1%	(−6.6%–8.8%)	0.779
	1–2 day	−0.2%	(−4.2%–3.8%)	0.924
	>2 day	Reference		
Weight at surgery	<1500 g	−5.4%	(−14.0%–3.2%)	0.218
	≧1500 g	Reference		
Surgical duration	≧120 min	5.7%	(1.3%–10.2%)	0.012
	<120 min	Reference		
ASA classification	≧3	−1.3%	(−5.7%–3.1%)	0.566
	<2	Reference		
Wound class	Clean-contaminated	−0.9%	(−6.0%–4.3%)	0.747
	Clean	Reference		
Past surgical history	yes	13.3%	(2.4%–24.1%)	0.017
	no	Reference		

*SSI* Surgical Site Infection, *ASA* American Society of Anesthesiologists.

There was no significant difference in risk when PAP was administered for ≤24 h vs. >24 h; the risk difference was −2.7% (95% confidence interval: −6.6% to 1.3%, *P* = 0.187). Similarly, no significant differences were observed when patients were grouped by PAP duration of ≤48 h vs. >48 h and ≤72 h vs. >72 h; the risk differences were 1.3% (95% confidence interval: −2.6% to 5.2%, *P* = 0.522) and 2.5% (95% confidence interval: −1.6% to 6.7%, *P* = 0.228), respectively.

Consistent results were obtained when additional confounders—institutions and surgical procedures—were included using IPW of the propensity scores. This analysis yielded a risk difference of −1.9% (for PAP duration ≤24 h vs. >24 h; 95% confidence interval: −7.2% to 3.4%, *P* = 0.482), with good covariate balance achieved (Supplement 5).

### Other risk factors for SSI

In the multivariable analysis using modified least-squares regression models, the following factors were significantly associated with an increased risk of SSI: operative time exceeding 120 min (risk difference: 5.7%, 95% confidence interval: 1.3% to 10.2%, *P* = 0.012), past surgical history (risk difference: 13.3%, 95% confidence interval: 2.4% to 24.1%, *P* = 0.017), and open surgery (risk difference: 5.9%, 95% confidence interval: 2.0% to 9.8%, *P* = 0.003) (Table 3).

## Discussion

This study retrospectively investigated PAP usage in 983 neonates who underwent surgery at multiple institutions and evaluated the relationship between the incidence of SSIs and the duration of PAP administration in neonatal surgery. A total of 950 patients (97%) received PAP, with approximately three quarters receiving it for ≥24 h. SSIs occurred in 91 of 983 patients (9%). In the multivariable analysis using a modified least-squares regression model and IPW of the propensity scores, no significant risk difference was observed when the PAP duration was ≤24 h compared with >24 h. However, a long operative time (≥120 min), past surgical history, and open surgery were significantly associated with an increased risk of SSI.

Previous studies of SSIs in neonatal surgery can be broadly divided into two main categories: those that investigated the incidence of SSIs and explored risk factors [22–26], and those that attempted to clarify the appropriate usage of PAP—both its type and duration [8, 9, 12, 13]. In this study, we addressed both themes by collecting a large volume of data related to perinatal factors, perioperative conditions, and antibiotic usage in neonates undergoing surgery. A unique feature of our study is the focus on “prophylactic” antibiotic use, achieved by excluding cases involving “therapeutic” antibiotic administration. While evidence on the incidence and risk factors of SSIs in neonatal surgery is gradually accumulating, data regarding the appropriate use of PAP remain limited. One major reason for this gap is that neonates often receive therapeutic antibiotics during the perioperative period [11]. For example, contaminated operations—such as those for intestinal perforation caused by immaturity of the intestinal tract—are more common in neonates. In such cases, antibiotics are used for treatment rather than prophylaxis, and prolonged administration is appropriate. Furthermore, because of their immunological immaturity, neonates are especially vulnerable to hospital-acquired infections such as urinary tract infections, pneumonia, and catheter-related bloodstream infections. As a result, antibiotic use often transitions from prophylactic to therapeutic. Previous studies [12, 13]—including those using large databases [6]—have not been able to clearly distinguish between prophylactic and therapeutic antibiotic use. By contrast, our study excluded cases involving therapeutic antibiotic administration for contaminated surgery or other infections. Therefore, we believe our analysis more accurately reflects the usage of *prophylactic* antibiotics in neonatal surgery.

In adult surgery, PAP administration typically does not exceed 24 h, except in special cases [1, 2]. In our study, even after excluding cases involving therapeutic antibiotic use, PAP was still administered for extended durations (>24 h) in many cases. This trend of prolonged administration is consistent with recent reports in neonatal surgery [12, 13].

Significant risk factors for SSIs identified in this study, such as a long operative time and past surgical history, have also been reported in previous studies on neonatal surgery [23, 26]. Other factors that are often considered clinically high risk for infection, such as low weight at surgery and short gestational age, did not show significant risk differences. Open surgery, another significant risk factor identified in our study, is also recognized as a risk factor in adult surgery [27]. However, there have been few laparoscopic or thoracoscopic procedures performed in neonates, and no prior studies have compared open surgery with laparoscopy as a risk factor for SSIs in this population. More cases involving laparoscopic surgery need to be accumulated to clarify whether laparoscopy or thoracoscopy is associated with the risk of neonatal SSI.

The incidence of SSIs in our study (9%) is comparable to that reported in previous studies of neonates, which range from 4.3% to 13.5% [22–26]. Although SSI rates vary widely depending on factors such as country, disease, and surgical procedure [2], the rate observed in our study appears higher than the recent overall rate in adults in Japan (5%) [28], but similar to the rate in the 2000s (approximately 10%). As SSI rates in adults have declined over time—partly due to improvements in perioperative management and the implementation of SSI surveillance—similar efforts, including the appropriate use of PAP, may contribute to a reduction in SSI rates in neonatal surgery as well.

Our study has important implications for the appropriate use of PAP. Regarding the duration of PAP, we categorized and compared durations of ≤24 h vs. >24 h, ≤48 h vs. >48 h, and ≤72 h vs. >72 h. In each comparison, shorter duration was not associated with an increased risk of SSI. Our findings are consistent with those of a previous study [8] on PAP in a mixed population of neonates and infants, which included 194 neonates and is often cited as evidence supporting short-term PAP in this population. Our study, which examined a larger cohort of 983 neonates, reached the same conclusion. We believe this is an important finding that supports and promotes the short and appropriate use of antibiotics in neonatal surgery.

This study has several limitations inherent to its design as a multi-institutional retrospective observational study without a unified perioperative protocol. First, we could not evaluate certain factors related to SSIs—such as preoperative skin preparation, timing of intraoperative antibiotic administration, and perioperative incision management—which may vary by institution or individual surgeon. Second, the participating institutions included a general hospital with a perinatal center, university hospitals, and children’s hospitals, introducing heterogeneity in patient backgrounds, although we adjusted for potential confounding factors identifiable from medical records. Third, both the type and duration of PAP were determined by the attending physician (pediatric surgeons or neonatologists) because there was no standardized protocol within each institution. Fourth, despite the large sample size, it was not possible to assess whether narrow-spectrum antibiotics were associated with SSI because broad-spectrum antibiotics were used in only a few cases. However, the incidence of SSI in this study was not higher than that reported in previous studies. Although broad-spectrum antibiotics are recommended for certain surgical procedures (e.g., sulbactam/ampicillin or tazobactam/piperacillin for esophageal anastomosis) [11, 29], narrow-spectrum antibiotics may be sufficient in neonates. Finally, the diagnosis of SSI relied on documentation in medical records, which may have led to overdiagnosis or underdiagnosis.

While antibiotic use clearly contributes to the prevention of SSIs, it is only one of many preventive measures, as noted in the CDC manual [2]. The lack of a consistent trend between the duration of PAP and the incidence of SSIs across facilities (Fig. 2) may also suggest that PAP administration alone is not sufficient and that comprehensive perioperative management is essential for SSI prevention. The next step in determining appropriate PAP use in neonates is to conduct a prospective observational study based on an antibiotic use protocol combined with unified, standardized perioperative management [30].

## Conclusion

This observational study examined the relationship between the incidence of SSIs and the duration of PAP in neonatal surgery. The results suggest that a short duration (<24 h) of PAP may not increase the risk of SSI. However, PAP was administered for relatively long periods in many cases, indicating that further efforts are needed to define and promote appropriate durations of PAP administration.

## Supplementary information

Legand of supplement

Supplement 1

Supplement 2

Supplement 3

Supplement 4

Supplement 5

## Data Availability

The datasets generated during/or analyzed during the current study and the code used to analyze and manage the data are available from the corresponding author on reasonable request.
