# Supplementary material for: Impact of prophylactic antibiotic duration on surgical site infection rate in neonatal surgery: a multicenter retrospective observational study
Source: J Perinatol. 2025 Aug 27;45(10):1443–9. doi: 10.1038/s41372-025-02400-3 (PMC12479342; doi:10.1038/s41372-025-02400-3)
Supplement: Supplementary file 1 — Legand of supplement [file 41372_2025_2400_MOESM1_ESM.docx]

**Supplement 1. Day of SSI Occurrence**

Bars show the number of SSI events by postoperative day. SSI, surgical site infection

**Supplement 2. Day of Duration of Perioperative Antibiotic Prophylaxis**

Bars show the number of cases by the duration of perioperative antibiotic prophylaxis.

**Supplement 3. Duration of Perioperative Antibiotic Prophylaxis Over Time**

Box plots display the duration of perioperative antibiotic prophylaxis for each year. The spline curve indicates the overall trend.

**Supplement 4. SSI Rate Over Time**

Bars represent the annual SSI rate in the full cohort. SSI, surgical site infection.

**Supplement 5. Standardized Mean Difference Plot**

To adjust for the additional confounders (institutions and surgical procedures), inverse probability weighting using propensity scores was applied. This helped to avoid overfitting due to the limited number of surgical site infections. Propensity scores were estimated via logistic regression including key covariates from the primary analysis, as well as institutions and surgical procedures. Balance of confounders after weighting was assessed visually using a standardized mean difference plot, with a standardized mean difference of <0.2 considered indicative of good balance. ASA, American Society of Anesthesiologists
