# Supplementary figures and images for: Impact of prophylactic antibiotic duration on surgical site infection rate in neonatal surgery: a multicenter retrospective observational study
Source: J Perinatol. 2025 Aug 27;45(10):1443–9. doi: 10.1038/s41372-025-02400-3 (PMC12479342; doi:10.1038/s41372-025-02400-3)

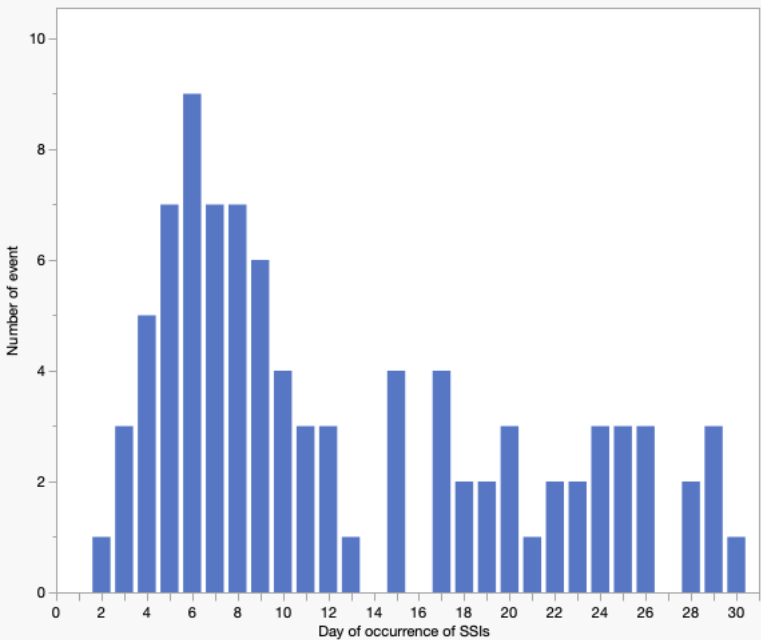

Supplement: Supplementary file 2 — Supplement 1 [file 41372_2025_2400_MOESM2_ESM.pdf]

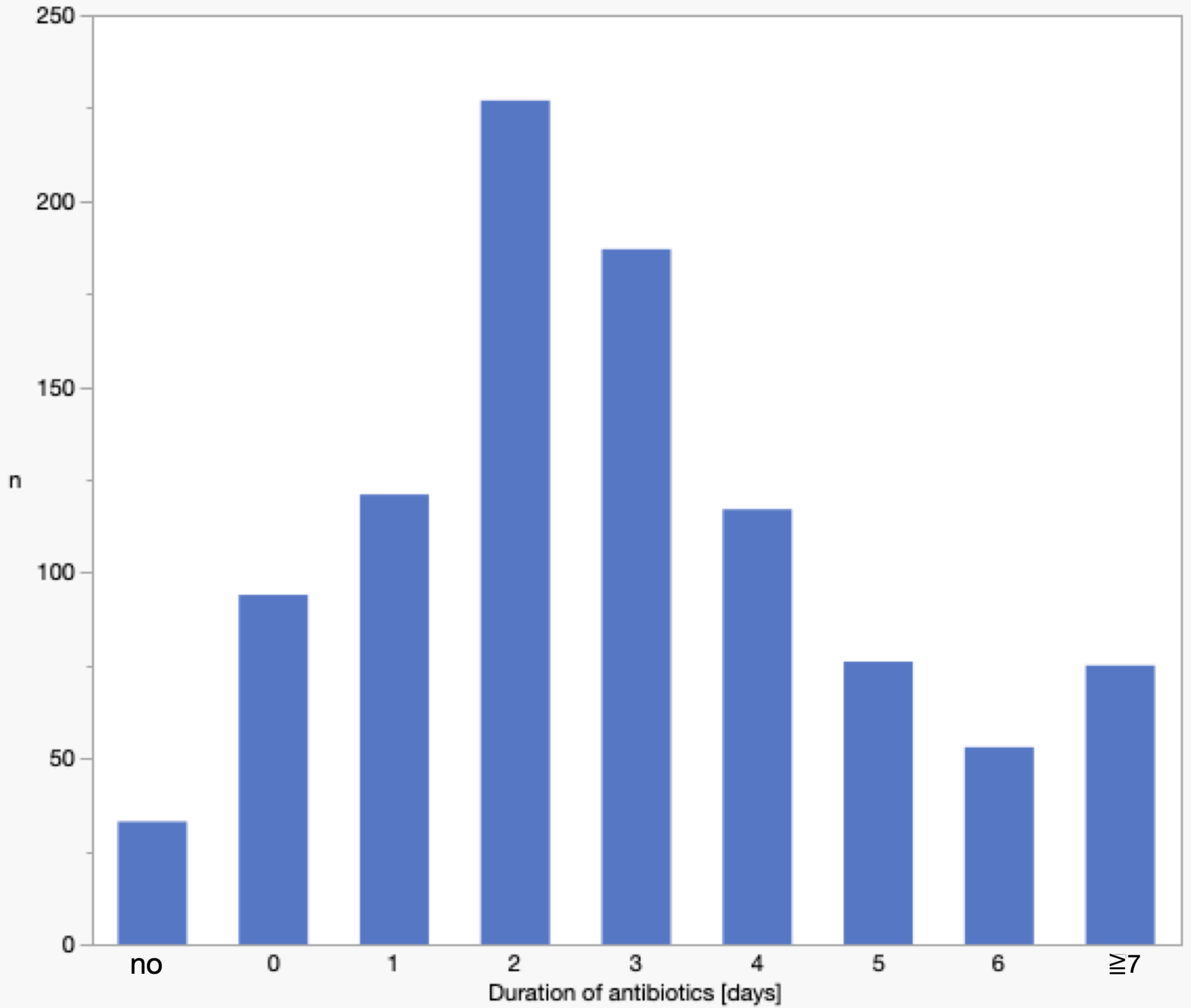

Supplement: Supplementary file 3 — Supplement 2 [file 41372_2025_2400_MOESM3_ESM.pdf]

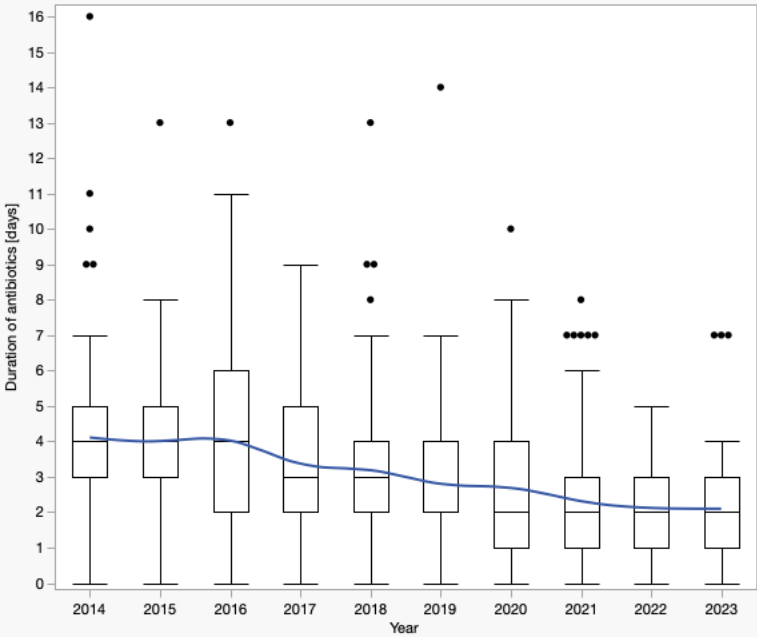

Supplement: Supplementary file 4 — Supplement 3 [file 41372_2025_2400_MOESM4_ESM.pdf]

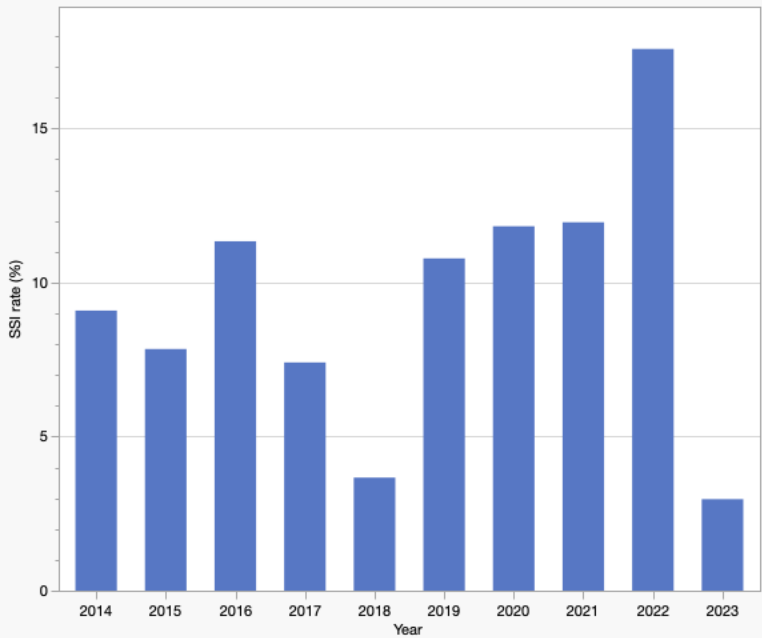

Supplement: Supplementary file 5 — Supplement 4 [file 41372_2025_2400_MOESM5_ESM.pdf]

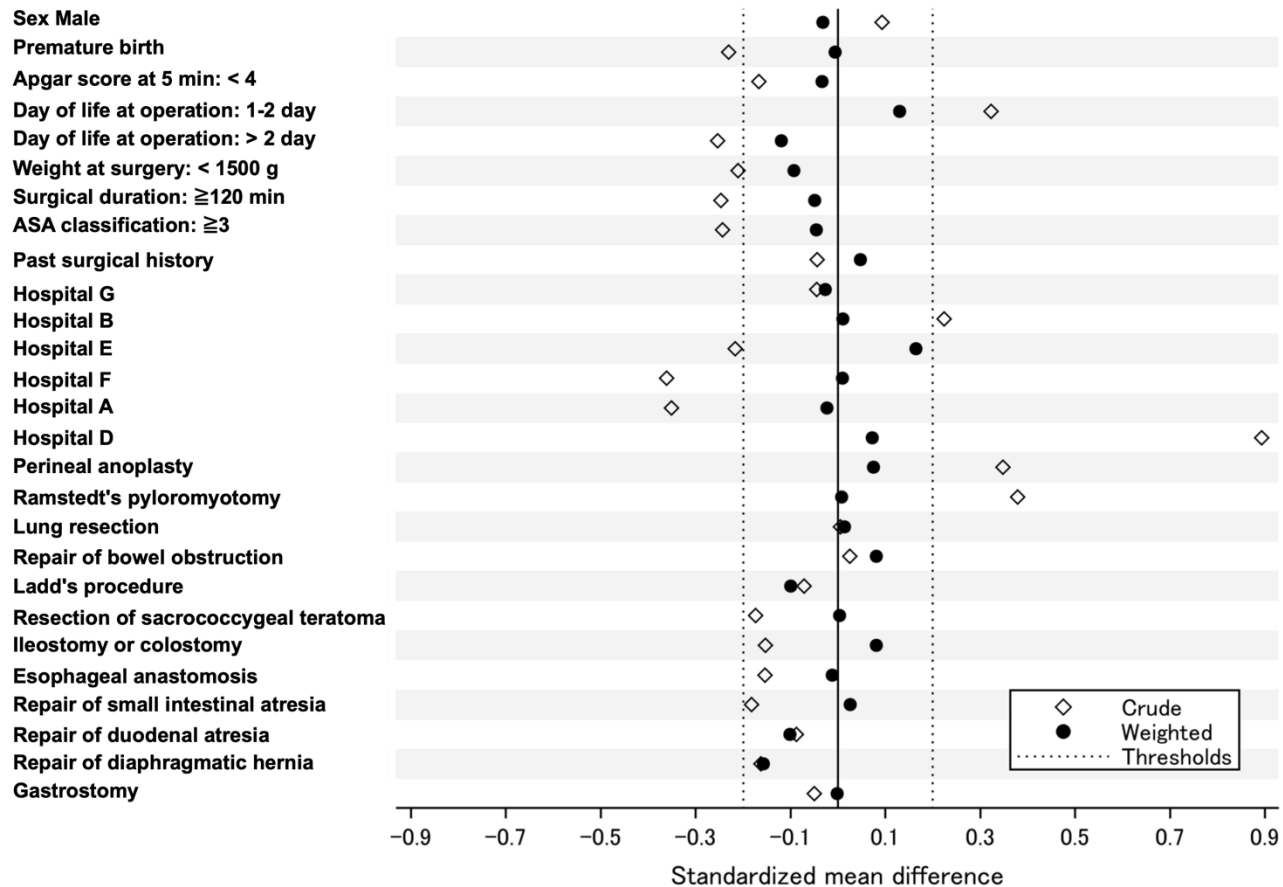

Supplement: Supplementary file 6 — Supplement 5 [file 41372_2025_2400_MOESM6_ESM.pdf]
